# Early synaptic deficits in the APP/PS1 mouse model of Alzheimer's disease involve neuronal adenosine A_2A_ receptors

**DOI:** 10.1038/ncomms11915

**Published:** 2016-06-17

**Authors:** Silvia Viana da Silva, Matthias Georg Haberl, Pei Zhang, Philipp Bethge, Cristina Lemos, Nélio Gonçalves, Adam Gorlewicz, Meryl Malezieux, Francisco Q. Gonçalves, Noëlle Grosjean, Christophe Blanchet, Andreas Frick, U Valentin Nägerl, Rodrigo A. Cunha, Christophe Mulle

**Affiliations:** 1 Interdisciplinary Institute for Neuroscience, University of Bordeaux, CNRS UMR 5297, F-33000 Bordeaux, France; 2 BEB PhD program CNC Coimbra, 3004-517 Coimbra, Portugal; 3 University of Bordeaux, Neurocentre Magendie, INSERM U862, F-33000 Bordeaux, France; 4 CNC-Center for Neuroscience and Cell Biology, University of Coimbra, 3004-517 Coimbra, Portugal; 5 Faculty of Medicine, University of Coimbra, 3004-504 Coimbra, Portugal

## Abstract

Synaptic plasticity in the autoassociative network of recurrent connections among hippocampal CA3 pyramidal cells is thought to enable the storage of episodic memory. Impaired episodic memory is an early manifestation of cognitive deficits in Alzheimer's disease (AD). In the APP/PS1 mouse model of AD amyloidosis, we show that associative long-term synaptic potentiation (LTP) is abolished in CA3 pyramidal cells at an early stage. This is caused by activation of upregulated neuronal adenosine A_2A_ receptors (A_2A_R) rather than by dysregulation of NMDAR signalling or altered dendritic spine morphology. Neutralization of A_2A_R by acute pharmacological inhibition, or downregulation driven by shRNA interference in a single postsynaptic neuron restore associative CA3 LTP. Accordingly, treatment with A_2A_R antagonists reverts one-trial memory deficits. These results provide mechanistic support to encourage testing the therapeutic efficacy of A_2A_R antagonists in early AD patients.

## Abstract (web summary)

Hippocampal synaptic dysfunctions are an early symptom of Alzheimer's disease. Here, the authors find adenosine A2A receptors are up-regulated in APP/PS1 model mice and that deleting or blocking receptor activity helps alleviate plasticity and memory impairments.

## Graphical abstract

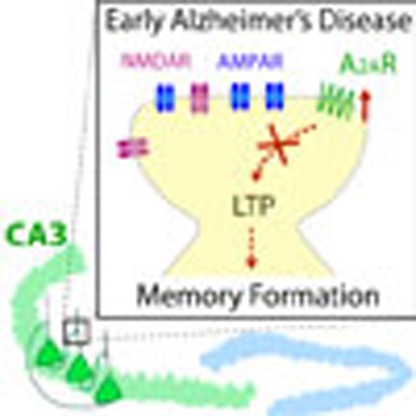

Loss of episodic hippocampal-dependent memory is the earliest clinical sign of Alzheimer's disease (AD), consistent with reduced activation of hippocampal regions during memory encoding tasks in patients with mild cognitive impairment^1^. Synaptic loss is the best morphological correlate of cognitive impairment in early AD, rather than amyloid-beta plaques, tangle formation or neuronal loss^2^. The CA3 subregion of the hippocampus encodes episodic memories, particularly at the earliest stage of acquisition, presumably by developing instant representations of a context^3^. The autoassociative network of recurrent connections among CA3 pyramidal cells (PCs) is thought to enable the storage of episodic memories through synaptic plasticity of these associative/commissural (A/C) inputs^4^,^5^. In mouse models of AD, synaptic dysfunction has been mostly studied in the CA1 region or dentate gyrus with very few studies addressing deficits in CA3 (ref. 6). Long-term potentiation (LTP) of synaptic transmission at Schaffer collateral-CA1 synapses is generally impaired in mouse models of AD (refs 6, 7). Whether synaptic plasticity is affected in the recurrent CA3 network in AD mouse models has not yet been addressed.

Dysregulation of NMDA receptors (NMDAR) has been proposed as a link between Aβ accumulation and disruption of LTP (ref. 8) although neuromodulation systems may also be impaired. Epidemiologic studies indicate that regular caffeine intake attenuates memory decline during aging^9^ and reduces the risk to develop AD (ref. 10). In animal models of AD, chronic caffeine intake prevents memory deterioration, an effect mimicked by the selective inhibition of A_2A_ receptors (A_2A_R), which are a main target of caffeine^11^. Conversely, the overactivation of hippocampal A_2A_R is sufficient to disrupt memory performance^12^. A_2A_R are upregulated in cortical areas of AD patients^13^ including in the hippocampal formation^14^, but the mechanisms by which the blockade of A_2A_R restores memory impairment are not understood. The impact of A_2A_R may depend on the stage of progression of the disease, with a role for astrocytic A_2A_R at late stages^14^. Here we provide evidence for early synaptic dysfunction in CA3 PCs in a mouse model of AD, and we explore the implication of NMDAR and A_2A_R.

## Results

### Impaired A/C synaptic plasticity in CA3 in APP/PS1 mice

**Figure 1:**
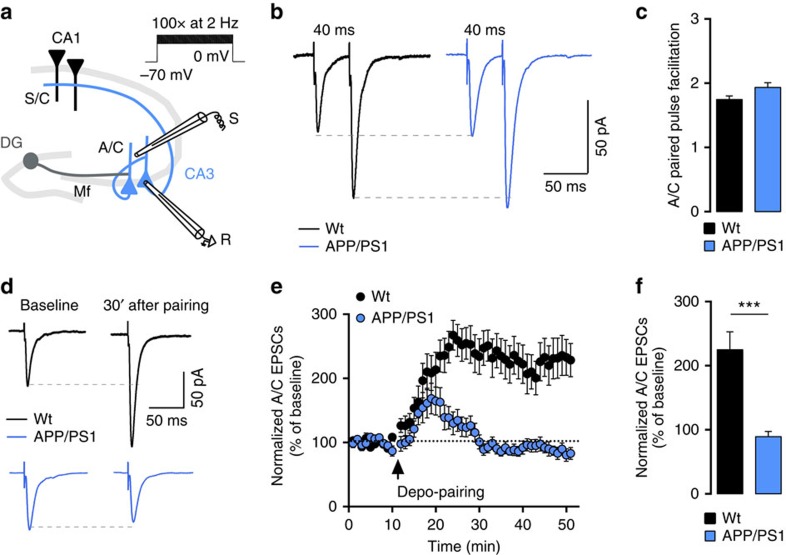
Early synaptic alterations in 6-month-old APP/PS1 CA3 PCs. (**a**) Scheme illustrating the hippocampus with a recording electrode on a CA3 PC (R) and a stimulating electrode (S) in the stratum radiatum. Depo-pairing protocol used to trigger NMDAR-dependent LTP: depolarization of postsynaptic cell to 0 mV paired with 100 stimuli at 2 Hz frequency. (**b**) Example traces of paired-pulse responses (40 ms interval, average of 5 sweeps) in cells from both genotypes. (**c**) Bar graph summarizing PPR values in wt (*n*=21) and APP/PS1 mice (*n*=25; *P*=0.054, unpaired *t*-test). (**d**) Sample traces representing average A/C-EPSCs 10 min before and 30 min after depo-pairing protocol. (**e**) Summary time course of normalized A/C-EPSCs in the experiments illustrated in **d**. (**f**) The robust LTP of A/C synapses found in 6-month-old wt mice (*n*=12) is abolished in APP/PS1 mice (*n*=11, ***P<0.0001, unpaired *t*-test; Supplementary Table 1). Recordings were performed in the presence of 10 μM bicuculline and 3 μM CGP55845.

We performed whole-cell patch clamp recordings of CA3 PCs to characterize the synaptic properties of A/C inputs in 6-month-old male APP/PS1 (Amyloid precursor protein (APP) gene with Swedish mutation and presenilin 1 gene (PS1) with deletion of exon 9) mice at early stages of amyloid-beta deposition^15^ when the CA3 region appears largely spared (Supplementary Fig. 1a). We initially recorded excitatory postsynaptic current (mEPSCs), which arise from the different types of glutamatergic synapses impinging on CA3 PCs. The average amplitude of mEPSCs was significantly decreased in APP/PS1 compared with wild-type (wt) mice (wt: 29±2 pA, APP/PS1: 23±1 pA, *P*=0.007), whereas mEPSC frequency, measured by the inter event interval (IEI), was only minimally affected (wt: 0.7±0.2 s, APP/PS1: 0.9±0.1 s; Supplementary Fig. 2a,b). The decreased amplitude was mainly attributed to mEPSCs with amplitudes <50 pA, suggesting that large amplitude mEPSCs arising from mossy fibre-CA3 synapses^16^ were not affected (Supplementary Fig. 2b). We further found that the paired-pulse ratio (PPR) of A/C synaptic responses was not different between APP/PS1 (1.9±0.1) and wt mice (1.8±0.1), arguing against presynaptic alterations (Fig. 1a–c). Pairing of presynaptic activation and postsynaptic depolarization induces an NMDAR-dependent LTP of A/C inputs in CA3 PCs (ref. 17). Pairing A/C stimulation (100 stimuli at 2 Hz) with a depolarization to 0 mV (Fig. 1a) induced a robust LTP of AMPA-EPSCs in wt mice (218±38%), which was absent in APP/PS1 mice (93±12%, *P*<0.0001; Fig. 1d–f).

**Figure 2:**
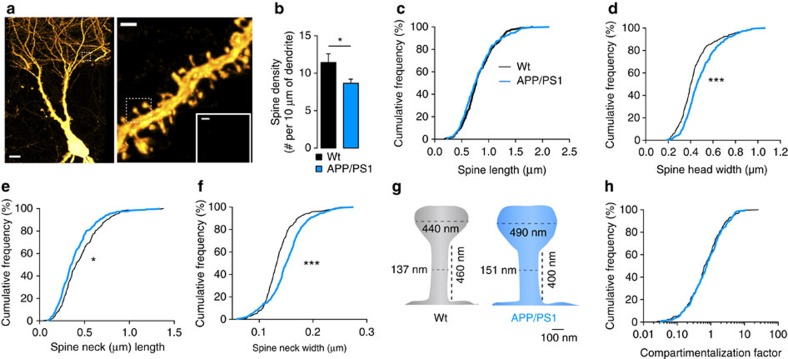
Alterations in A/C spine morphology. (**a**) Representative STED images obtained from RABVΔG-GFP(RG) infected neurons. Scale bar, left panel, 10 μm; right panel, 1 μm; inset, 250 nm. (**b**) Bar graph summarizing the reduction of spine density. Several branches of distal dendrites of CA3 PCs were analysed in wt (*n*=8 branches, 4 mice) and in APP/PS1 mice (*n*=9 branches, 4 mice; *P=0.037, unpaired *t*-test). (**c**) Cumulative distribution illustrating the absence of any difference in spine lengths between both genotypes (Kolmogorov–Smirnov (KS) test *P*=0.144). Number of spines analysed was equal for graphs from **c** to **h**, *n*=300 for wt and *n*=288 for APP/PS1, from four mice for each genotype. (**d**) Cumulative distribution of spine head width (KS-test ***P<0.0001), (**e**) spine neck length (KS-test *P=0.015) and (**f**) spine neck width (KS-test ***P<0.0001) from wt and APP/PS1 mice. (**g**) Schematic spines illustration of morphological alterations observed in A/C spines with mean values indicated. (**h**) The cumulative distribution of the compartmentalization factor calculated for each spine was not different between wt (1.35±0.13) and APP/PS1 mice (1.35±0.10, *P*=0.063, KS-test).

**Figure 3:**
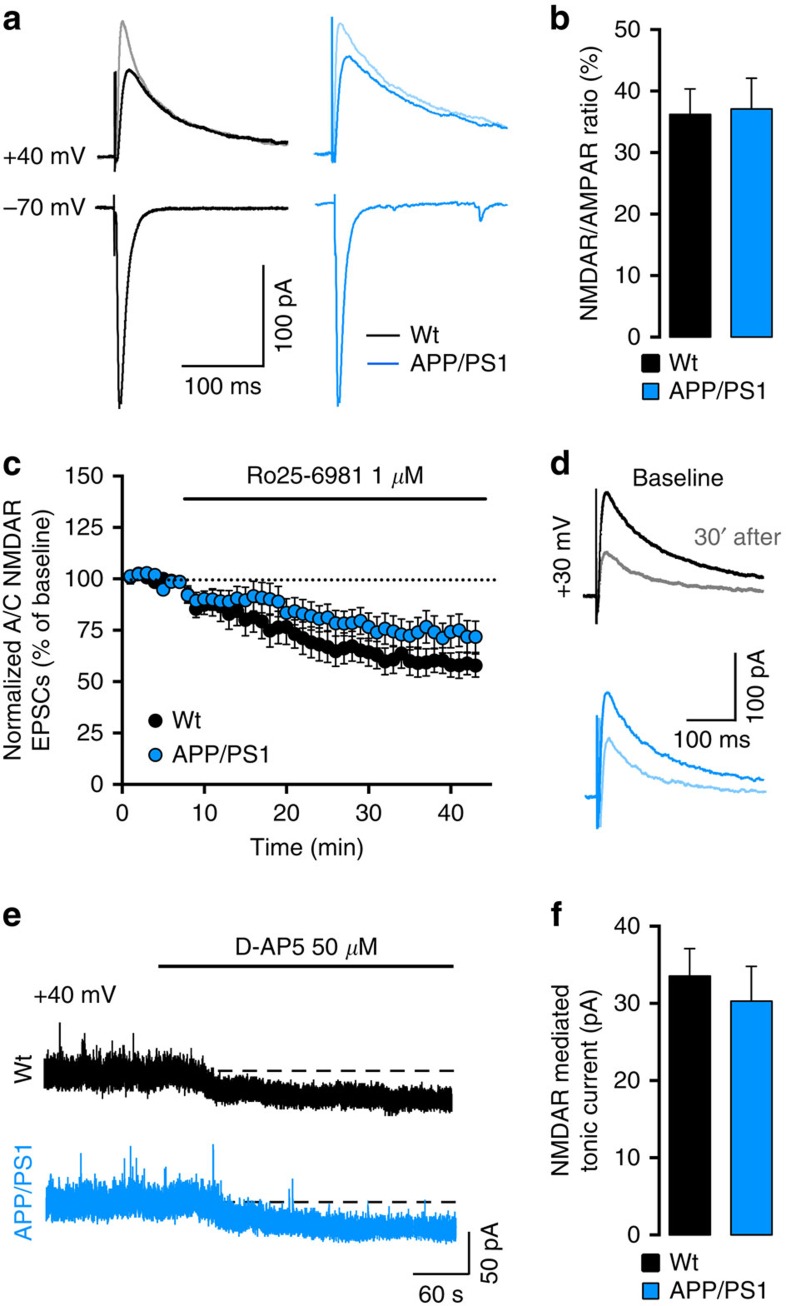
Loss of A/C LTP is not associated with dysregulation of NMDAR. (**a**) A/C EPSCs were recorded at negative and positive potentials, and NMDAR-EPSCs were pharmacologically isolated with 20 μM NBQX (darker traces at +40 mV). Traces represent the average of 40 sweeps recorded at 0.1 Hz. (**b**) Mean values of NMDAR/AMPAR ratios obtained with isolated currents at −70 mV (AMPAR) and +40 mV (NMDAR) from wt (*n*=18) and APP/PS1 mice (*n*=16; *P*=0.894, unpaired *t*-test). (**c**) Summary plot of the time course of A/C NMDAR-EPSCs amplitude during the application of Ro25-698 in wt (*n*=13) and APP/PS1 cells (*n*=16, *P*=0.155, unpaired *t*-test). Amplitude of A/C NMDAR-mediated EPSCs was normalized to the mean amplitude of an 8 min baseline period and quantification was performed after 30 min of drug application. (**d**) Average traces of pharmacologically isolated (20 μM NBQX) A/C NMDAR-EPSCs obtained at a stimulation frequency of 0.1 Hz during baseline and after 30 min of Ro25-698 (1 μM) application (lighter traces). (**e**) Representative examples of the shift in holding current observed in CA3 PCs clamped at +40 mV in response to D-AP5 revealing the tonic activation of extrasynaptic NMDAR at rest. AMPAR and KAR were blocked with 20 μM NBQX. (**f**) Summarizing bar graph representing the shift of inward current in wt (*n*=14) and APP/PS1 cells (*n*=14, *P*=0.573, unpaired *t*-test). Data calculated as the difference between the last min of baseline and the last min after a 6-min D-AP5 application. Recordings were performed in the presence of 10 μM bicuculline and 3 μM CGP55845.

To explore the mechanisms underlying the abolition of LTP of A/C inputs in APP/PS1 mice, we first tested whether this loss was correlated with morphological alterations of CA3 dendritic spines. Bilateral stereotaxic injections of retrograde rabies virus expressing green fluorescent protein (GFP; RABV)^18^ into CA1 were performed to specifically label CA3 PCs. We quantified spine density and morphology in these neurons using stimulated emission depletion (STED) microscopy. We found a decrease in the density of spines in the *stratum radiatum* of APP/PS1 mice (wt: 11.5±1.1 spines per 10 μm, APP/PS1: 8.7±0.6 spines per 10 μm, *P*=0.037; Fig. 2a,b). STED microscopy allowed us to examine traditionally neglected key nanoscale features of spine morphology (Supplementary Table 2). Although spine length was similar in both genotypes (wt: 0.82±0.02 μm, APP/PS1: 0.81±0.02 μm, Fig. 2c), we found a marked shift to larger spine heads in APP/PS1 mice (wt: 0.44±0.01 μm, APP/PS1: 0.49±0.01 μm, *P*<0.0001, Fig. 3d), in parallel with shorter and wider spine necks (Fig. 2e,f and Supplementary Table 2). As synapse compartmentalization is strongly shaped by spine morphology^19^, these changes (Fig. 2g) could potentially explain the decreased ability of the pairing protocol to induce LTP. However, the compartmentalization factor, which is a measure of biochemical compartmentalization of spine synapses (see definition in the Methods section), was preserved because the effects of the structural changes cancelled each other out (Fig. 2h). Thus the morphological phenotype seems insufficient to explain the absence of LTP observed at A/C synapses in APP/PS1 mice.

### Loss of A/C LTP is not associated with alterations of NMDAR

We tested whether dysregulation of NMDAR function, which has been implicated in impaired synaptic plasticity in models of AD (ref. 8), may be causally related to the loss of LTP in APP/PS1 mice. Insufficient membrane potential depolarization can be ruled out as a possible cause for the loss of A/C LTP in APP/PS1 mice as our LTP protocol controls for postsynaptic membrane potential. We found no difference in synaptic NMDAR/AMPAR ratio between wt (36±4%) and APP/PS1 mice (37±5%; Fig. 3a,b). However, the relative expression of different GluN2 NMDAR subunits at synapses may strongly modulate plasticity^20^, and the toxic effects of amyloid-beta oligomers applied in cultured neurons and acute slices is thought to involve the GluN2B subunit^21^,^22^. We found no difference in the inhibition of NMDAR EPSC amplitude by Ro25-6981 (1 μM), a selective antagonist of GluN2B-containing NMDAR (wt: 59.0±5.9%, APP/PS1: 73.6±7.6%; Fig. 3c,d) or in NMDAR EPSC decay time (Supplementary Fig. 3a,b), ruling out a major change in GluN2 subunit composition of synaptic NMDAR in CA3 PCs in APP/PS1 mice. The subcellular localization of NMDAR (synaptic versus extrasynaptic) leads to the activation of different intracellular signalling pathways; importantly, extrasynaptic NMDAR were shown to be essential for amyloid-beta mediated toxicity^23^,^24^. We evaluated extrasynaptic NMDAR by measuring the amplitude of tonic NMDAR-mediated currents recorded at +40 mV. Tonic currents were blocked to the same extent by the NMDAR antagonist D-AP5 (50 μM) in both genotypes (wt: 34±4 pA and APP/PS1: 30±5 pA; Fig. 3e,f). In addition, the blockade of tonic currents by Ro25-6981 (1 μM) did not indicate any change in GluN2B content (Supplementary Fig. 3c,d). Finally, we reasoned that if dysregulation of NMDAR was responsible for the loss of A/C LTP in APP/PS1 mice, then the selective potentiation of synaptic NMDAR by D-serine^25^ may rescue plasticity. Enhancement of synaptic NMDAR by bath application of 10 μM D-serine (∼20% increase in current amplitude, Supplementary Fig. 3e–g) did not rescue A/C LTP in APP/PS1 mice (109.0±9.5%, Supplementary Fig. 3h–j and Supplementary Table 1) or alter A/C LTP in wt mice. Hence, the complete loss of A/C LTP in 6-month-old APP/PS1 mice does not appear to correlate with alterations in the function or GluN2B content of neither synaptic nor extrasynaptic NMDAR.

### Inhibition of A2AR rescues A/C LTP in APP/PS1 mice

**Figure 4:**
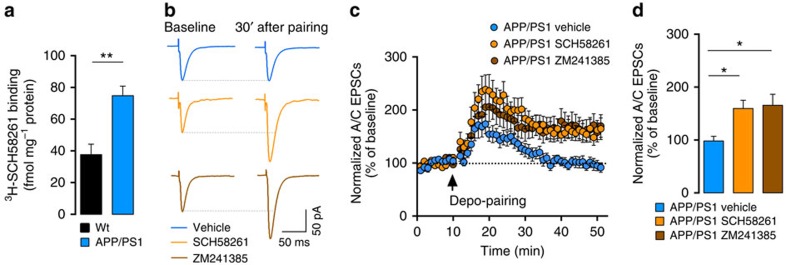
Blockade of neuronal A_2A_R restores synaptic plasticity in CA3 PCs. (**a**) The density of A_2A_R is increased in synaptic membranes prepared from the CA3 region of APP/PS1 mice compared with wt littermates (*n*=6, **P=0.002, Mann–Whitney test). (**b**) Example traces representing a 10 min average of A/C-EPSCs before and 30 min after depo-pairing LTP protocol in the presence of SCH58261 (50 nM) or ZM241385 (50 nM). (**c**) Summary time course of normalized A/C-EPSCs recorded from APP/PS1 mice during LTP protocol performed in the presence of the two different A_2A_R antagonists or in control conditions. (**d**) Bar graph representing mean LTP amplitude recorded at 30–40 min after depo-pairing protocol represented in **c**. A 10-min incubation period with two different classes of A_2A_R antagonists, SCH58261 (*n*=11) and ZM241385 (*n*=12, *P*=0.006 Kruskal–Wallis test with Dunn's multiple comparison test), rescued the LTP of A/C synapses in APP/PS1 mice (*n*=11 for vehicle, Supplementary Table 1). Electrophysiology recordings were performed in the presence of 10 μM bicuculline and 3 μM CGP55845.

A_2A_R control synaptic plasticity^12^, are involved in memory impairment^11^,^26^ and are upregulated in the brain of AD patients^13^,^14^ and animal models of AD^27^,^28^. We used a binding assay on isolated CA3 synaptic membranes and showed a robust increase of A_2A_R density in 6-month-old APP/PS1 mice (wt: 38±7 fmol per mg protein; APP/PS1: 75±6 fmol per mg protein, *P*=0.002; Fig. 4a). Based on these results, we investigated if a short incubation of the slices for 10 min with the selective A_2A_R antagonist SCH58261 (50 nM) could affect synaptic plasticity at A/C synapses. Strikingly, SCH58261 rescued in large part A/C LTP in APP/PS1 synapses (160±16%), in comparison with APP/PS1 slices treated with vehicle solution (98±9%, Fig. 4b–d). ZM241385 (50 nM), a chemically distinct and selective A_2A_R antagonist was equally effective in rescuing A/C LTP (wt: 165.9±20.8%, *P*=0.063; Fig. 4d and Supplementary Table 1). The difference in mEPSCs amplitude observed between APP/PS1 and wt littermates (wt: 25.2±1.5 pA; APP/PS1: 19.5±1.1 pA) was not rescued by a short incubation with SCH58261 (wt_SCH_: 24.0±1.5 pA; APP/PS1_SCH_: 19.6±0.8 pA; *P*=0.006 for genotype effect; *P*=0.173 for drug effect, Supplementary Fig. 4a–c). Similarly, the distribution of mEPSCs amplitudes in both genotypes was not altered by SCH58261 (Supplementary Fig. 4b). Although SCH58261 incubation caused a small alteration in the distribution of IEIs between mEPSCs (Supplementary Fig. 4d), it did not cause any significant effect on the mean IEI values (wt_Baseline_: 0.4±0.1 s; wt_SCH58261_: 0.6±0.2 s; APP/PS1_Baseline_: 0.6±0.2 s; APP/PS1_SCH58261_: 0.8±0.2 s, Supplementary Fig. 4e).

**Figure 5:**
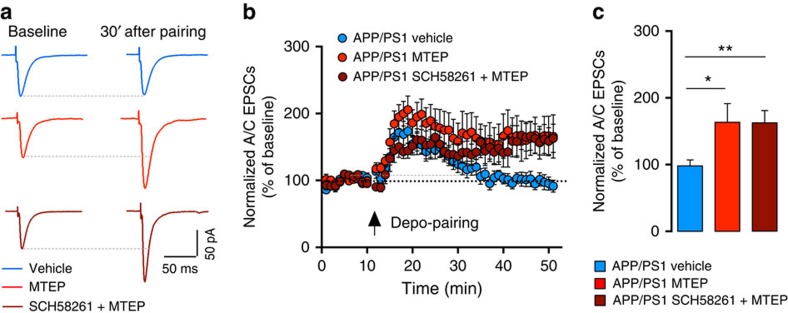
A_2A_R and mGluR5 are involved in the loss of A/C LTP in APP/PS1 mice. (**a**) Example traces representing the average of A/C-EPSCs 10 min before and 30 min after depo-pairing LTP protocol. Recordings were performed in the presence of 10 μM bicuculline and 3 μM CGP55845. (**b**) Summary time course of normalized A/C-EPSCs recorded from APP/PS1 mice during LTP protocol performed in the presence of MTEP (10 μM) and MTEP+SCH58261 (50 nM). (**c**) Bar graph representing mean LTP amplitude recorded at 30–40 min after depo-pairing protocol represented in **b**. A 10-min incubation with MTEP (*n*=12) or MTEP+SCH58261 (*n*=11, *P*=0.008 Kruskal–Wallis test with Dunn's multiple comparison test) rescued the LTP of A/C synapses in APP/PS1 mice (*n*=11 for vehicle group, Supplementary Table 1).

A_2A_R act synergistically with mGluR5 in hippocampal neurons^29^, and mGluR5 antagonists rescue contextual fear conditioning in APP/PS1 mice^30^. Accordingly, we found that a selective antagonist of mGluR5 (MTEP, 10 μM) also rescued A/C LTP levels in APP/PS1 mice to values similar to those obtained with the A_2A_R antagonist SCH58261 (164±28%, Fig. 5a–c). A combined incubation with both MTEP and SCH58261 did not further increase A/C LTP levels in 6-month-old APP/PS1 mice (163±18%, *P*=0.008, Fig. 5a–c and Supplementary Table 1), suggesting that A_2A_R and mGluR5 operate through a common pathway to impair LTP. Importantly, these antagonists did not further increase the level of A/C LTP in wt mice (Supplementary Fig. 5a–c and Supplementary Table 1). Pharmacological inhibition of either A_2A_R or mGluR5 did not fully rescue A/C LTP, possibly because the antagonists attenuate LTP in wt mice (Supplementary Fig. 4e,f). Adenosine neuromodulation depends on a balanced activation of inhibitory A_1_R and A_2A_R. We tested whether adenosine A_1_R levels were comparatively affected by using a binding assay on purified CA3 synaptic membranes and observed a modest increase of A_1_R density in 6-month-old APP/PS1 mice (wt: 938.2±27.2 fmol per mg protein; APP/PS1: 1044.0±19.0 fmol per mg protein, *P*=0.002; Supplementary Fig. 6a). To test if this alteration in A_1_R density affected A/C synapses, we recorded evoked AMPAR EPSCs and bath applied 100 nM DCPCX (a selective A_1_R antagonist). DCPCX equally increased the amplitude of AMPAR EPSCs in APP/PS1 (134.0±11.0%) and wt mice (143.3±17.8%, Supplementary Fig. 6b–d), arguing for similar levels of A_1_R at those synapses.

**Figure 6:**
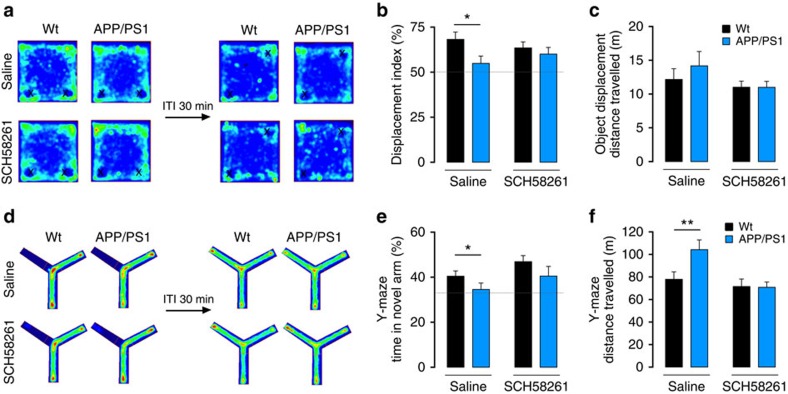
Treatment with A_2A_R blockers restores one-trial memory impairment in APP/PS1 mice. (**a**) Average occupation plot representing the pattern of exploration of the mice in the object displacement test. Mice were treated with SCH58261 or saline for 6 days (**b**) Bar graph representing the percentage of time spent by mice exploring the displaced object. The displacement index is higher in wt_saline_ (*n*=12) when compared with APP/PS1_saline_ (*n*=9, *P=0.034, two-way ANOVA, genotype effect; Sidak's multiple comparisons test wt_saline_ versus APP/PS1_saline_, *P=0.0374). By contrast, whereas SCH58261 is devoid of effects in wt (*n*=11, Sidak's multiple comparisons test wt_saline_ Saline versus wt_SCH58361_, *P*=0.594), it reverted the lower percentage of time that APP/PS1 mice interacted with the novel object (*n*=10, Sidak's multiple comparisons test APP/PS1_saline_ versus APP/PS1_SCH58361_, *P*=0.6047). (**c**) No alterations were found in the average distance travelled by mice (wt_Saline_: 12.2±1.6 m, *n*=12, APP/PS1_Saline_: 14.5±1.9 m, *n*=9, *P*=0.405; wt_SCH58261_: 11.0±0.9 m, *n*=11, APP/PS1_SCH58261_: 11.0±0.9 m, *n*=10, *P*=0.096, two-way ANOVA). (**d**) Average occupation plot of exploration in the Y-maze task. (**e**) Bar graph representing the percentage of time spent in the novel arm by wt and APP/PS1 mice treated with SCH58261 or saline for 7 days. When comparing APP/PS1 (*n*=9) and wt (*n*=12) mice in the saline groups, we observed a difference in the percentage of time spent in the novel arm (*P=0.024, two-way ANOVA, genotype effect; Sidak's multiple comparisons test wt_saline_ versus wt_SCH58361_, *P=0.0347). SCH58261 treatment had no effect in wt groups (*n*=11, Sidak's multiple comparisons test wt_saline_ versus wt_SCH58361_, *P*=0.901), but reverted the memory deficit in APP/PS1 mice (*n*=10, Sidak's multiple comparisons test APP/PS1_saline_ versus APP/PS1_SCH58361_, *P*=0.410) (**f**) Distance travelled in the test phase of the modified Y-maze (wt_Saline_: 77.9±6.7 m, *n*=12, APP/PS1_Saline_: 104.2±8.6 m, *n*=9; wt_SCH58261_: 71.5±6.7 m, *n*=11, APP/PS1_SCH58261_: 70.8±4.8 m, *n*=10, **P=0.006, two-way ANOVA with Sidak's multiple comparisons test). Horizontal dashed lines in **b** and **e** represent the random displacement values for better visualization.

A_2A_R antagonists can prevent Aβ-induced memory impairment in mice^26^. We thus tested whether SCH58261 (intraperitoneal injection, 0.1 mg per kg, for 6–7 days) could revert deficits in one-trial memory tasks, which depend on CA3 circuits^3^,^4^, in a different group of 6-month-old APP/PS1 mice. When tested in the object displacement paradigm (30 min inter-trial interval), APP/PS1 mice showed impaired recognition of a displaced object, which was rescued on A_2A_R inhibition (Fig. 6a–c). Wt mice treated with saline displayed a higher displacement index (68.2±4.0%), whereas APP/PS1 mice did not show any preference for the displaced object (54.9±4.1%, *P*=0.034 for genotype effect, Fig. 6b). This difference between genotypes was abrogated by SCH58261 treatment (wt_SCH58261_; 63.5±3.3%; APP/PS1_SCH58261_: 60.0±3.7%). Likewise, A_2A_R inhibition rescued the performance of APP/PS1 mice in a modified Y-maze task (30 min inter-trial interval), as shown by the increase in the percentage of time exploring the novel arm (wt_Saline_ 40.5±2.4 and APP/PS1_Saline_: 34.6±2.8%, *P*=0.024 for genotype effect, Fig. 6d–f). APP/PS1 mice treated with SCH58261 showed no difference in the time spent in the novel arm (42.7±4.1%) when compared with wt mice treated with SCH58261 (46.9±2.7%, Fig. 6e).

### A_2A_R expressed in CA3 PCs are involved in A/C LTP deficits

**Figure 7:**
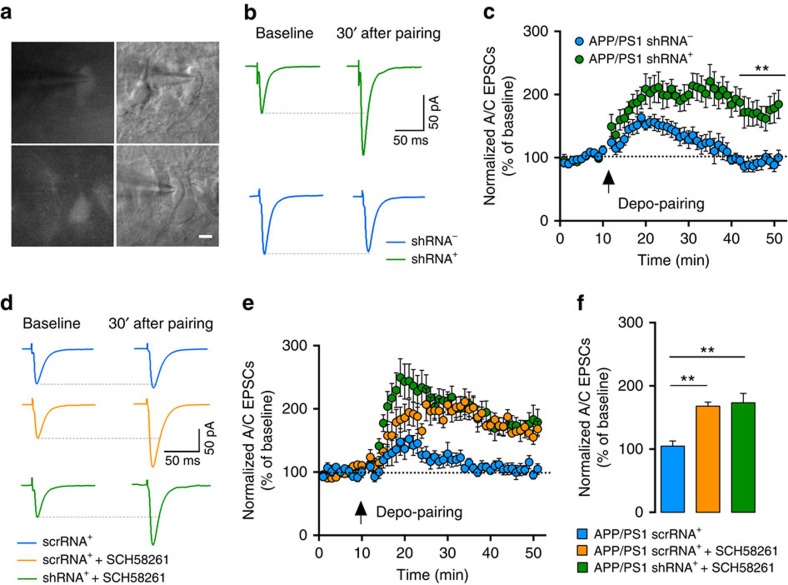
Genetic silencing of neuronal A_2A_R restores A/C LTP. (**a**) Example pictures of CA3 PCs infected with shRNA lentivirus. Infected cells (shRNA^+^) and non-infected cells (shRNA^−^) in the proximity of infected cells were patched for the experiments in **b**. Scale bar represents 10 μm. (**b**) Example traces representing average A/C-EPSCs 10 min before and 30 min after LTP protocol in infected and non-infected cells. (**c**) Summary time course of normalized A/C-EPSCs during depo-pairing LTP protocol, recorded from APP/PS1 mice injected with A_2A_R shRNA; in shRNA^+^ cells there was a rescue of the A/C LTP (*n*=7) that was absent in shRNA^−^ cells (*n*=7, **P=0.001, Mann–Whitney test, Supplementary Table 1). (**d**) Example traces representing the average of A/C-EPSCs 10 min before and 30 min after depo-pairing LTP protocol in infected cells with A_2A_R shRNA or srcRNA lentivirus. (**e**) Summary time course of normalized A/C-EPSCs recorded from APP/PS1 mice during LTP protocol performed in scrRNA^+^ cells (*n*=9) in the absence and scrRNA^+^ (*n*=7) or shRNA^+^ cells (*n*=9) in the presence of SCH58261 (50 nM). (**f**) Bar graph representing mean LTP amplitude recorded at 30–40 min after depo-pairing protocol represented in **e**. APP/PS1 cells infected with scrRNA virus (scrRNA^+^) do not sustain LTP (Supplementary Table 1). A 10-min incubation period with SCH58261 increased LTP amplitude of scrRNA^+^ cells to levels similar to the genetic silencing of A_2A_R (Supplementary Table 1). In shRNA^+^ cells, SCH58261 did not further increase the LTP levels (**P=0.0014, Kruskal–Wallis test with Dunn's multiple comparison test between groups). All electrophysiology recordings were performed in the presence of 10 μM bicuculline and 3 μM CGP55845.

Since both neuronal^12^ and astrocytic A_2A_R (ref. 14) may control memory performance, we sought to understand if the pharmacological rescue of plasticity at early stages of AD is either due to an action on neurons or glia. For this purpose, we employed a knockdown approach of A_2A_R in neurons using a VSV-G-coated lentivirus expressing shRNA against A_2A_R and a fluorescent marker (shA_2A_R, see validation in Supplementary Information). One month after stereotaxic infection in the CA3 region of 5-month-old APP/PS1 mice, a few GFP-positive CA3 PCs (shA_2A_R^+^, expressing the shRNA) could be identified in hippocampal slices (Fig. 7a). To check for neuronal tropism of the lentiviral construct, we injected eGFP-shA_2A_R lentivirus in the CA3 region of wt mice and performed immunohistochemistry on hippocampal sections. We observed a near exclusive co-localization with the neuronal marker NeuN for virally injected eGFP-positive cells, whereas no co-localization was observed with the astrocytic marker glial fibrillary acidic protein (GFAP) (Supplementary Fig. 7).

We found that genetic silencing of A_2A_R in individual CA3 PCs rescued LTP of A/C synapses in these cells in 6-month-old APP/PS1 mice. LTP of A/C synapses did not recover in uninfected neighbouring sh-A_2A_R^−^ CA3 PCs (shA_2A_R^+^: 174±20%, shA_2A_R^−^ cells: 92±9%, *P*=0.001; Fig. 7a–c) or in cells infected with scramble RNA (scrRNA^+^: 105±8%, Fig. 7d–f). A/C LTP could be rescued in scrRNA^+^ cells with a short SCH58261 incubation as in control APP/PS1 CA3 PCs (168±6%, *P*=0.0014, Fig. 7d–f). Furthermore, the extent of A/C LTP following SCH58261 incubation was not different between scrRNA^+^ and shRNA^+^ cells (173±15%), indicating that the A_2A_R shRNA occludes the effect of SCH58261 in shRNA^+^ cells and not in control neurons (Supplementary Table 1 and Fig. 7f). This strongly suggests that the loss of A/C LTP is caused by gain of function of A_2A_R specifically in neurons and not glial cells at this early stage of AD. In addition, removal of A_2A_R from a single postsynaptic CA3 PC is sufficient to rescue A/C LTP, strongly suggesting a pathological function of postsynaptic A_2A_R.

## Discussion

This study provides the first characterization of AD-related synaptic impairment in the autoassociative network of recurrent connections between CA3 PCs which is crucially involved in the initial encoding of memory^3^,^5^. At early stages of AD pathology in APP/PS1 mice, we found no major alteration of basal AMPAR or NMDAR-mediated transmission. However, we show that associative pairing between presynaptic A/C stimulation and postsynaptic spiking failed to induce LTP in single CA3 PCs.

Studies of LTP in mouse models of AD have often provided contradictory results^6^, possibly depending on the age and on the experimental conditions to induce plasticity. In CA1, LTP of field EPSPs induced by high-frequency stimulation is attenuated—but not abolished—in an age-dependent manner in 4–6-month-old APP/PS1 mice^31^. Spine shape may determine the ability of synapses to undergo LTP by tuning biochemical and electrophysiological compartmentalization^19^. We observed a moderate decrease of spine density, which may correlate with subtle differences in the frequency of mEPSCs but cannot explain the abolition of LTP. Using STED microscopy, we unravelled subtle changes in spine necks and spine heads in 6-month-old APP/PS1 mice. However, as the morphological changes are predicted to leave biochemical synapse compartmentalization intact, it is unlikely that differences in synapse morphology can account for the LTP defect in APP/PS1 mice.

Associative LTP of A/C synapses depends on the activation of NMDAR during the induction phase^17^,^32^. Many studies have proposed NMDAR and in particular GluN2B-containing NMDAR as targets for Aß peptides^8^. GluN2B is thought to be important for induction of LTP, due to the direct physical interaction of its C-terminal tail with CaMKII (ref. 33). Our electrophysiological results indicate that the loss of A/C LTP in APP/PS1 mice cannot be attributed to decreased function of synaptic NMDAR or to a major change of GluN2B-containing NMDAR. We found no change in tonically activated extrasynaptic NMDAR, which were proposed to participate in the inhibition of LTP by soluble Aß oligomers^23^. In line with our data, chronic blockade of GluN2B-containing NMDAR does not rescue behaviour or spine morphology in PS2APP mice^34^. We do not exclude the possibility that NMDAR signalling may be strongly compromised at later stages of AD. However our study suggests that NMDAR may not be a valid target to restore hippocampal synaptic plasticity and the moderate cognitive deficits at early stage of AD pathology, in support of the lack of clinical efficiency of memantine in mild AD (ref. 35).

Strikingly, we found that the acute inhibition of A_2A_R by two different antagonists rescued LTP of A/C synapses, strongly suggesting that activation of A_2A_R by ambient adenosine or by adenosine produced during the LTP induction protocol disrupts synaptic plasticity in APP/PS1 mice. This raises several questions with regards to the source of adenosine, the cellular localization of A_2A_R and the signalling mechanism engaged to compromise LTP. On brain injury, A_2A_R undergo increased expression in both neurons and glia^14^,^36^; their conditional removal from astrocytes ameliorates spatial memory selectively at later stages of AD (ref. 14). Our pharmacological experiments do not allow distinguishing effects of the antagonists on glial cells, pyramidal neurons or interneurons. However, we found that A_2A_R are upregulated in CA3 synapses at early stages of AD. Moreover, silencing A_2A_R selectively in an individual CA3 PC is sufficient to restore A/C LTP. Thus, the increase of A_2A_R in postsynaptic CA3 PCs can by itself explain the abolition of LTP. Although we cannot rule out an increased expression of astrocytic A_2A_R at this stage, this is not required for impaired plasticity. Interestingly, the prevention of synaptic plasticity can be relieved by acute antagonism of A_2A_R, providing some potential therapeutic strategies for early cognitive dysfunction in AD. Accordingly, the treatment of APP/PS1 mice with SCH58261 improved their one-trial spatial memory performance in both an object displacement and a modified Y-maze task, which is thought to depend on the activity of CA3 circuits^3^. More work is necessary to establish clear links between impaired A/C LTP and rapid acquisition of memory. Systemic administration of SCH58261 did not affect recall in one-trial memory tasks in wt mice, although it partly inhibited A/C LTP. Conversely, in APP/PS1 mice, behavioural deficits were markedly improved by SCH58261 administration, although A/C LTP deficit was only partly reverted.

The mechanism by which activation of upregulated A_2A_R compromises LTP is unknown. Because silencing A_2A_R in an individual CA3 PC rescues LTP, postsynaptic A_2A_R are likely at play. A_2A_R are pleiotropic receptors activating multiple G proteins and transducing pathways; the extended C-terminal domain of A_2A_R engages several proteins other than those canonically involved in signalling by G-protein coupled receptors^37^. Furthermore, the signalling pathways of upregulated A_2A_R in disease-like conditions may be altered^38^. Which of these multiple transducing systems is associated with the control by A_2A_R of synaptic plasticity is still unknown.

The ability of both A_2A_R and mGluR5 to disrupt A/C LTP is intriguing and may provide a clue to the underlying mechanism^29^. Interestingly, A_2A_R and mGluR5 are both also necessary for the expression of LTP of NMDARs in CA3 PCs (ref. 39). The molecular mechanisms by which the amount of A_2A_R is increased at early stages in neurons and at later (neuroinflammatory) stages in astrocytes still remains to be deciphered, in view of the complexity of the A_2A_R gene promoter^40^. Upregulation of A_2A_R at early stages may not be restricted to CA3 PCs and may include interneurons and interneuronal connections to regulate the overall excitability of CA3 circuits^41^. Nevertheless, postsynaptic A_2A_R in CA3 PCs, rather than such possible alterations account for deficits in A/C CA3 LTP.

Overall, the present study shows that at the early onset of AD-like features in APP/PS1 mice, associative long-term synaptic plasticity is abolished in CA3 PCs due to the activation of upregulated A_2A_R in the postsynaptic compartment rather than to modifications of synaptic structure or NMDAR function. Our results are based on one-mouse model of AD, and it would certainly be important in the near future to extend this to other experimental models of cognitive deficits. Nonetheless, the exquisite ability of A_2A_R blockade to restore the defective A/C CA3 LTP in APP/PS1 mice, in parallel to studies linking A_2A_R to cognitive deficits^11^,^12^, provides an additional mechanistic support to encourage testing the therapeutic efficacy of A_2A_R antagonists in early AD patients.

## Methods

### Mice

APP/PS1 mice were obtained from Jackson's Lab and used according to regulations of the University of Bordeaux/CNRS Animal Care and Use Committee. The colony was maintained in a hemizygote state by crossing transgenic female mice to B6C3F1/J male mice. Throughout all their life, littermate wt and APP/PS1 male mice were housed in groups ranging from 4 to 10 animals per cage with free access to water and food. The large transparent plexiglas cages were kept in a temperature-regulated room on a 12-h light/dark cycle, and protected from exterior pathogens by a filter. All experiments were performed in the light phase of the circadian cycle in 6 months APP/PS1 and age-matched wt littermates.

### Electrophysiology

Mice were anaesthetized with a ketamine (75 mg kg^−1^) and xylazine (10 mg kg^−1^) mix and intracardially perfused with ice-cold oxygenated cutting solution composed of 200 mM of sucrose, 20 mM glucose, 0.4 mM CaCl_2_, 8 mM MgCl_2_, 2 mM KCl, 1.3 mM NaH_2_PO_4_, 26 mM NaHCO_3_, 1.3 mM ascorbate, 0.4 mM pyruvate and 3 mM kynurenic acid (pH 7.3). When the solution coming out of the heart was free of blood, mice were decapitated and the head immersed in ice-cold cutting buffer. The brain was quickly dissected and parasagittal slices (350 μm thick) were cut using a Leica vibratome (Leica VT 1200S) in the same solution. The slices were kept for 20 min in an oxygenated resting artificial cerebrospinal fluid (aCSF) containing 110 mM NaCl, 2.5 mM KCl, 0.8 mM CaCl_2_, 8 mM MgCl_2_, 1.25 mM NaH_2_PO_4_, 26 mM NaHCO_3_, 0.4 mM ascorbate, 3 mM pyruvate and 14 mM glucose (pH 7.3) at 33 °C in the presence of kynurenic acid (2 mM). The slices were then transferred into another resting aCSF, now without kynurenic acid and left at room temperature for a maximum of 6 h after cutting. All drugs from Sigma-Aldrich, unless otherwise stated (see Supplementary Table 3 for details).

Cells were identified by differential interference contrast microscopy using an Olympus fixed stage upright microscope (BX51WI) equipped with a × 40 magnification immersion objective. Once at the electrophysiology set-up, slices were superfused with oxygenated (95% O_2_, 5% CO_2_) aCSF composed by 125 mM NaCl, 2.5 mM KCl, 2.3 mM CaCl_2_, 1.3 mM MgCl_2_, 1.25 mM NaH_2_PO_4_, 26 mM NaHCO_3_ and 14 mM glucose (pH 7.4) and with 10 μM bicuculline. A period of 10 min was allowed for slice stabilization and removal of the excess Mg^2+^ from the resting solution. Whole-cell recordings were made at room temperature from visually identified CA3 PCs with borosilicate glass capillaries with 3–5 MΩ resistance filled with 140 mM CsMSO_3_, 2 mM MgCl_2_, 4 mM NaCl, 0.2 mM EGTA, 5 mM phosphocreatine, 3 mM ATPNa_2_, 0.4 mM GTP and 10 mM HEPES (pH 7.2). The access resistance was <20 MΩ and the experiment was discarded if it changed by >20%. EPSCs were evoked using a patch pipette (∼5 MΩ) filled with aCSF positioned in the CA3 stratum radiatum. The identification of the A/C synaptic currents was performed according to the following criteria: (1) frequency facilitation not >1.2 when switching stimulation from 0.1 to 1 Hz, (2) paired-pulse facilitation ratio <3 and (3) EPSCs decays free of a secondary peak that might indicate the presence of polysynaptic contamination. No liquid-junction potential correction was used. Recordings were made using an EPC10.0 amplifier (HEKA Elektronik), filtered at 0.5–1 kHz and analysed using IGOR Pro and Neuromatic V2.6 softwares.

### Stereotaxic injections and viral vectors

A small hairpin (sh) RNA was engineered to target the mouse A_2A_R (shA_2A_RNA), with the following sequence: 5′-CTA GTT TCC AAA AAG AAC AAC TGC AGT CAG AAA TCT CTT GAA TTT CTG ACT GCA GTT GTT-3′ and 5′-CGG GGA TCT GTG GTC TCA TAC AGA AC-3′. These oligomers and the H1 forward primer 5′-CAC CGA ACG CTG ACG TCA TCA ACC CG-3′ were used for PCR with the pBC-H1 plasmid (pBC; Stratagene, Amsterdam, The Netherlands) containing the H1 promoter (GenBank: X16612, nucleotides 146–366) as a template. The silencing H1-shRNA cassettes were inserted into the lentivector plasmid SIN-cPPT-PGK-EGFP-WHV-LTR-TRE-RFA together with an EGFP reporter gene. As a control, we engineered a lentivector expressing a scrambled sequence of the RNA targeting A_2A_R (scrRNA). Both the shA_2A_RNA and scrRNA were packaged in VSV-G-coated lentiviral vectors. Lentivectors were produced in HEK293T cells, with a four-plasmid system, as previously described^42^ and the lentiviral particles content was determined by assessing HIV-1 p24 antigen levels (Gentaur, Spain). Viral stocks were stored at −80 °C until use and were thawed on ice before their *in vivo* administration. For control experiments, the lentivectors were administered to mice anaesthetized with avertin (240 μg g^−1^, i.p.) and placed in a stereotaxic frame (Stoelting, Wood Dale, USA). To test the *in vivo* efficiency of the shA_2A_RNA, we first injected the lentivector (286,000 ng of p24 antigen per ml) unilaterally into the mouse striatum, where the high density of A_2A_R allows a faithful quantification of A_2A_R downregulation. Mice received 1.4 μl of shA_2A_RNA or scrRNA (0.2 μl per min) with an automatic injector (Stoelting) in the following coordinates: antero-posterior: +0.6 mm; medio-lateral: ±1.8 mm; and ventral: −3.3 mm. After 3 weeks, an immunohistochemical analysis of the percentage striatal area labelled with eGFP (ref. 43) showed that the lentivectors transfected 27.2±2.7% (*n*=4) of the volume of dorsal striatum. This caused a 68±8% (*n*=4) decrease of A_2A_R mRNA expression, evaluated by quantitative PCR (ref. 44) and a 55±6% (*n*=4) decrease of A_2A_R protein density in the striatum, as evaluated by Western blot analysis^45^. Mice were kept in their home cages for 4 weeks before being killed for preparation of slices and recording of transfected and non-transfected neurons, identified by the presence or absence of eGFP. The estimated amount of cells transfected varied from experiments to experiments and was ∼15 cells per slice and 600 cells per infection (similar profile to Supplementary Fig. 6).

The GFP expressing RABV were stereotaxically injected as previously described^46^ in CA1 stratum radiatum and the cellular CA3 morphology was analysed after 6 days. For STED microscopy, we performed immunohistochemistry (anti-GFP Alexa 488) on 60 μm brain sections to further increase the signal to background ratio.

### Immunohistochemistry

(a) For immunolabelling performed on RABV infected brains, coronal sections (60 μm) were washed three times with 0.5% Triton-X100 (TX) in 1 × PBS (20 min each) and pre-incubated 1 h in a serum solution (goat serum 10%+0.5% TX in 1 × PBS). The sections were washed three times with 1 × PBS and immersed in the primary antibody solution (rabbit anti-GFP antibody A11122, Invitrogen; 1/1,000 in 0.5% TX in 1 × PBS) for 24 h at 4 °C on a shaker. After washing four times with 1x PBS, the sections were incubated with the secondary antibody (goat anti-rabbit Alexa Fluor 488 antibody, A11008 from Invitrogen; 1/1,000 diluted in 1 × PBS+0.5% TX) for 2 h at room temperature. The brain sections were mounted in ProLong Gold Antifade mounting medium (Invitrogen/Molecular Probes) and covered by individual round glass coverslips of 18-mm diameter (0.17±0.01 mm thick). (b) Immunolabelling for detection of amyloid plaques was performed as described above, using a primary antibody raised against human amyloid-beta peptides (polyclonal rabbit antibody, 1/500; ABCAM ab2539) and goat anti-rabbit secondary antibody coupled with Alexa Fluor 488. (c) Immunohistochemistry to determine the cell types infected in the hippocampal CA3 area was performed on sections from lentivirus-infected mice as described above, using following antibodies: anti-NeuN (mouse Millipore MAB377, 1/400), anti-GFAP (rabbit Millipore AB5804, 1/1,000), donkey anti-mouse secondary antibody coupled Alexa Fluor 594 (1/200) and donkey anti-rabbit secondary antibody coupled Alexa Fluor 594 (1:200).

### STED microscopy and morphometric analysis

Super-resolved images of spine morphology were obtained with a custom-built STED microscope with a nominal spatial resolution of 50 nm. All images were acquired in the stratum radiatum above the CA3b region as stacks of 10 *z*-sections with a step size of 192 nm and a pixel size of 40 nm × 40 nm. For spine counts and density calculations over large field-of-views (image from Fig. 2a left and data Fig. 2b), a home-built two-photon microscope, (*λ*_exc_=920 nm) equipped with a water-immersion objective (60X LUMFI, 1.1 NA, Olympus) was used as previously described^47^. For more detailed analyses of spine morphology we used a home-built STED microscope. It was based on an inverted microscope (DMI 6000 CS Trino, Leica), using a pulsed-laser diode (PDL 800-D, Picoquant) for excitation (*λ*_exc_=485 nm, ∼90 ps) and a Ti:Sa-OPO system (Chameleon, Coherent; OPO, APE) for fluorescence quenching (λ_STED_=595 nm, ∼200 ps), as described previously^48^. The pulses of originally 200 fs duration were stretched to ∼300 ps by dispersion via a 100-m-long polarization-preserving fiber (Schäfter+Kirchhoff, Hamburg, Germany). To create the STED focal doughnut, a polymeric phase plate (RPC Photonics, Rochester, NY) was introduced into the path of the expanded STED beam, imprinting a helical phase ramp onto the wavefront. The STED and excitation pulses were synchronized via external triggering of the laser diode, and the delay was adjusted with a custom-built electronic delay generator. Both beams were overlapped with a dichroic mirror (AHF Analysentechnik, Tübingen, Germany), and focused onto the fixed sample by an oil objective (HCX PL APO, 1.47 NA, × 100; Leica, Wetzlar, Germany). A telecentric beam scanner (Yanus IV, TILL Photonics, Gräfelfing, Germany) with scan and tube lenses from the microscope manufacturer was used to steer the beam. Focusing by the objective was controlled via a piezo actuator (P-721 PIFOC, PI Physik Instrumente, Karlsruhe, Germany). The fluorescence was collected episcopically using a dichroic mirror and a 525/50 band-pass filter, and imaged onto a multimode optical fiber connected to an avalanche photodiode (SPCM-AQR-13-FC, PerkinElmer, Waltham, MA). Images were acquired using ImSpector software (courtesy of A. Schönle, Max Planck Institute for Biophysical Chemistry, Göttingen, Germany). All morphological measurements were done using a custom-made plug-in for ImageJ where the spine length was measured from the base of the dendrite to the edge of the head, following the curvature of the spine neck. Spine neck width is reported as the average from multiple spine neck profiles, drawn orthogonal to the spine neck curvature. Spine head width was measured orthogonal to the axis of the spine neck. The length and width of each spine neck was determined by measuring the full width at half-maximum of a Gaussian fit applied to line profiles across the spine neck.

To evaluate the impact of nanoscale alterations in spine morphology on diffusional coupling, we calculated the compartmentalization factor, which is defined as:


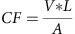


where *V* is the spine head volume, *L* the spine neck length and *A* the cross-sectional area of the spine neck. compartmentalization factor corresponds to the time constant *τ* of diffusional recovery 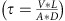 after a step change in concentration of a molecule with a diffusion coefficient *D*, serving as a quantitative measure of the degree of biochemical compartmentalization of a synapse^49^.

### Binding assays

On collection, the two hippocampi from each mouse were stored at −80 °C. The hippocampi were then unfrozen in ice-cold Krebs solution (140 mM NaCl, 5 mM KCl, 25 mM HEPES, 1 mM EDTA, 10 mM glucose and pH 7.4) and sliced in a McIlwain chopper (800 μm). Each individual slice was then placed on a rubber surface, over a drop of ice-cold Krebs solution, and CA3 subslices were manually dissected under microscope magnification^50^. To purify synaptic membranes, the CA3 subslices from each animal were placed in an eppendorf with 500 μl of ice-cold sucrose solution (0.32 M) containing 50 mM Tris–HCl, 2 mM EGTA and 1 mM dithiothreitol, pH 7.6, homogenized with a micro-Potter-Elvehjem and centrifuged at 3,000*g* for 10 min at 4 °C. The supernatants were collected, centrifuged at 14,000*g* for 20 min at 4 °C and the pellet was resuspended in 500 μl of a 45% (v/v) Percoll solution made up in a Krebs solution. After centrifugation at 14,000*g* for 2 min at 4 °C, the top layer was removed (synaptosomal fraction), washed in 500 μl of Krebs solution and vigorously resuspended in 500 μl of Tris/Mg solution (50 mM Tris and 10 mM MgCl_2_, pH 7.4). The mixtures were centrifuged at 30,000*g* for 30 min to pull-down synaptic membranes and resuspended in 500 μl of Tris/Mg solution with 4 U ml^−1^ adenosine deaminase (Sigma), to remove endogenous adenosine. We have previously shown that the synaptic membranes provide an enrichment of markers of nerve terminals (SNAP-25, syntaxin-I) and of the postsynaptic density (PSD-95), which display a <2% contamination with astrocytic material, as gauged by the western blot evaluation of GFAP in the purified synaptosomes used to prepare synaptic membranes^51^.

To evaluate the binding density of A_2A_R, we used ^3^H-SCH58261 {2-(2-Furanyl)-7-(2-phenylethyl)-7H-pyrazolo[4,3-e][1,2,4]triazolo[1,5-c]pyrimidin-5-amine}, which binds selectively cortical A_2A_R (ref. 52), whereas the binding density of A_1_R was determined using ^3^H-1,3-dipropyl-8-cyclopentylxanthine (DPCPX). Binding of ^3^H-SCH58261 (specific activity of 77 Ci mmol^−1^; prepared by Amersham and generously offered by Dr Ennio Ongini, Shering-Plough, Milan, Italy) or of ^3^H-DPCPX (specific activity of 102.1 Ci mmol^−1^; DuPont NEN, Boston, MA, USA) was for 2 h at room temperature with 6.5–13.5 μg ml^−1^ of synaptosomal protein in a final volume of 200 μl in Tris/Mg solution containing 4 U ml^−1^ adenosine deaminase with a single supra-maximal and selective concentration of ^3^H-DPCPX (10 nM) or of ^3^H-SCH58261(6 nM). Specific binding was determined by subtraction of the non-specific binding, which was measured in the presence of 1 μM 8-{4-[(2- aminoethyl)amino]carbonylmethyl-oxyphenyl}xanthine (XAC), a mixed A_1_/A_2A_R antagonist. Each binding assay data point was performed in duplicate. The binding reactions were stopped by vacuum filtration through glass fibre filters (B filters) using a 12-wells-Millipore harvester. The filters were then placed in scintillation vials and 4 ml of scintillation liquid cocktail (Aquasafe 500 Plus Ready Safe) was added. Radioactivity was determined after at least 12 h with a counting efficiency of 55–60% using a Tricarb 2900TR liquid scintillation analyser (Perkin Elmer). After subtraction of the non-specific from the total binding, the disintegration per min values corresponding to the specific binding were converted into absolute amounts fmol of bound ^3^H-SCH58261 or ^3^H-DPCPX, which was normalized per mg of protein, quantified with the BCA method.

### Behavioural experiments

The behavioural tasks, namely the modified Y-maze and object location tasks, were performed as a measurement of short-term spatial memory (to focus on CA3 region) and based on the innate preference of rodents to explore novelty. All the behavioural tests were performed during the light phase of the circadian cycle (between 08:30–02:00 hours), under red/low light (12lx). The intraperitoneal injection of SCH58261 was performed around 06:00 hours, to avoid the repercussion of the acute effect of the drug in the behavioural tasks and in the animal sleeping cycle. Between each trial the apparatus was cleaned with a 10% ethanol solution to avoid odour cues. At the end of the training trial, the mice were removed from the apparatus and kept in an individual cage during the inter-trial intervals. Mice were first tested in the object displacement test and then tested 12 h later in the modified Y-maze test, based on our previous validation that there is no influence of the object displacement test on the modified Y-maze test with this temporal interval either in control mice or in mice with memory deficits (Rial *et al.*^53^). All experimental data was analysed using the ANY-maze video tracking system, Stoelting, US.

*Modified Y-maze*. The Y-maze apparatus consisted of three arms (42 cm long, 15 cm height and 8 cm large) made from impermeable black formica with a brown floor and was placed at the ground level. During the training phase, one arm was blocked by a removable door. In this phase the mice were positioned in the start arm, facing the center of the maze and allowed to explore only two arms (start and other) for 8 min. The test was performed 30 min later (with the door blocking the novel arm removed) and the animal were placed again in the start arm and allowed to explore the three arms during 8 min. The number of entries and the time spent in each arm was measured, as previously described^53^,^54^.

*Object displacement/object location*. The object displacement protocol used was carried out as previously described^53^,^54^. Briefly, the mice were first habituated for a 10-min period in the empty apparatus, a box composed by transparent plexiglass walls and brown floor (50 cm wide, 50 cm deep and 40 cm high), placed at the ground level. In the training phase, performed 24 h after the habituation phase, two identical plastic 50 ml Falcon tubes filled with dyed sterile water were placed 15 cm away from the walls of the arena. The mice were placed at the center of the apparatus and allowed to explore these two identical objects for 10 min, and we recorded the time spent sniffing/whisking or looking (≤1 cm) at the objects. The test was performed 30 min later, where one of the objects was moved creating a different spatial combination for the animal to explore during 5 min; again the time spent exploring the objects in the novel and familiar locations was recorded. All locations for the objects were counterbalanced among the groups. The displacement index was determined by the percentage of the time spent exploring the displaced object (*T*_novel_) over the time spent exploring both objects. Displacement index is defined as (*T*_novel_ × 100)/(*T*_novel_+*T*_familiar_).

SCH58261 injections (0.1 mg kg^−1^, i.p.) were done each day, for 7 consecutive days before behavioural evaluation. Electrophysiological experiments were not carried out on animals used for behavioural experiments.

### Statistical analysis

All statistical analyses were performed with Prism6 GraphPad Software. Values are presented as mean±s.e.m. in the text and figure legends. The data normality was tested using the D'Agostino & Pearson omnibus normality test. If data was normally distributed, a Student's *t*-test or one-way and two-way analysis of variance (ANOVA; to compare more than two independent groups) was performed; otherwise, a Mann–Whitney test was used (to compare two groups) and Kruskal–Wallis test followed by a Dunn's multiple comparison test (to compare more than two groups). Within-cell comparisons were made with Wilcoxon matched pairs test using non-normalized values. Data distributions were analysed using the Kolmogorov–Smirnov test with the data from wt mice as reference. Statistical differences were considered significant at *P*<0.05.

### Data availability

The data that support the findings of this study are available from the corresponding author on request.

## Additional information

**How to cite this article:** Viana da Silva, S. *et al.* Early synaptic deficits in the APP/PS1 mouse model of Alzheimer's disease involve neuronal adenosine A_2A_ receptors. *Nat. Commun.* 7:11915 doi: 10.1038/ncomms11915 (2016).

## Supplementary Material

Supplementary Information Supplementary Figures 1-7 and Supplementary Tables 1-3
