# Supplementary material for: Early synaptic deficits in the APP/PS1 mouse model of Alzheimer's disease involve neuronal adenosine A2A receptors
Source: Nat Commun. 2016 Jun 17;7:11915. doi: 10.1038/ncomms11915 (PMC4915032; doi:10.1038/ncomms11915)
Supplement: Supplementary Information — Supplementary Figures 1-7 and Supplementary Tables 1-3 [file ncomms11915-s1.pdf]

SUPPLEMENTARY INFORMATION

SUPPLEMENTARY FIGURES

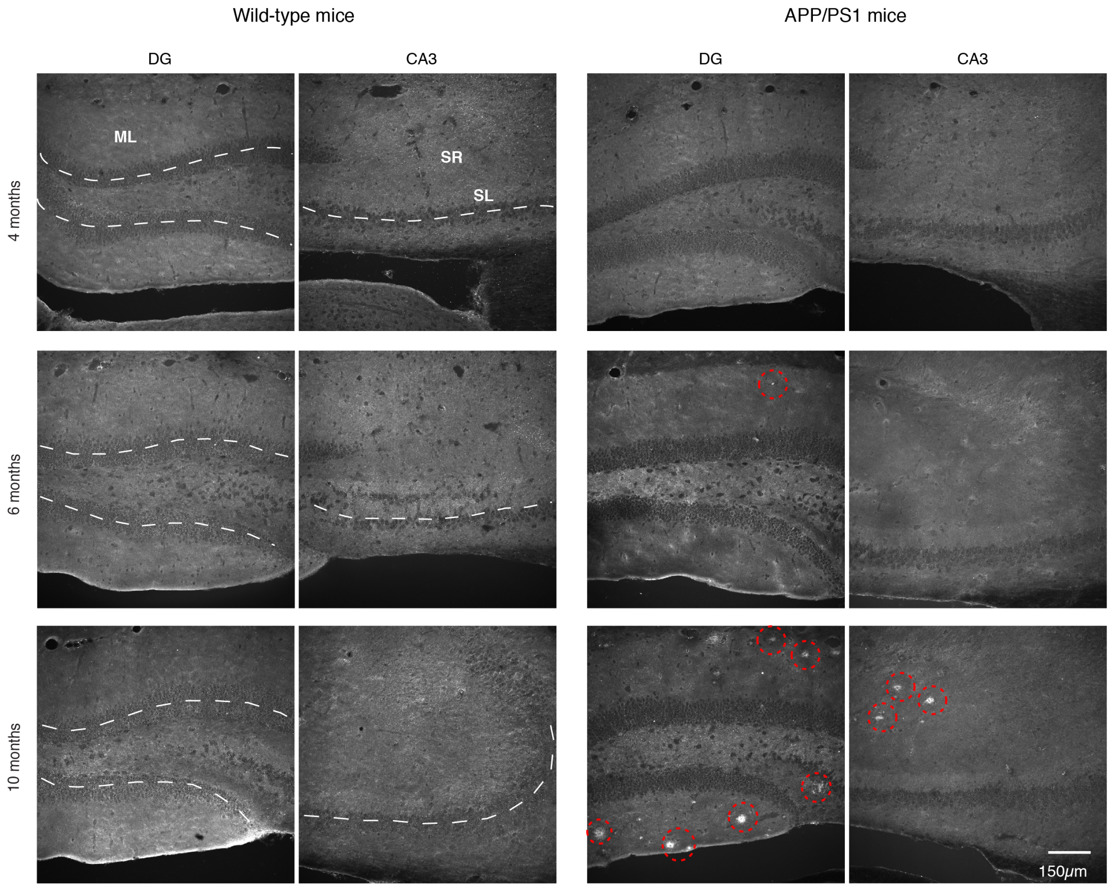

Supplementary Figure 1

Representative immunohistochemistry of Aβ insoluble deposits in the hippocampus of APP/PS1 mice. At 4 and 6 months, Aβ plaques are absent from the CA3 region and only in rare cases (n = 1 slice out of 40, from 4 APP/PS1 mice) present in the molecular layer (ML) of the DG. After 10 months numerous plaques were visible in the ML and *stratum radiatum* (SR). We never observed Aβ plaques in the *stratum lucidum* (SL).

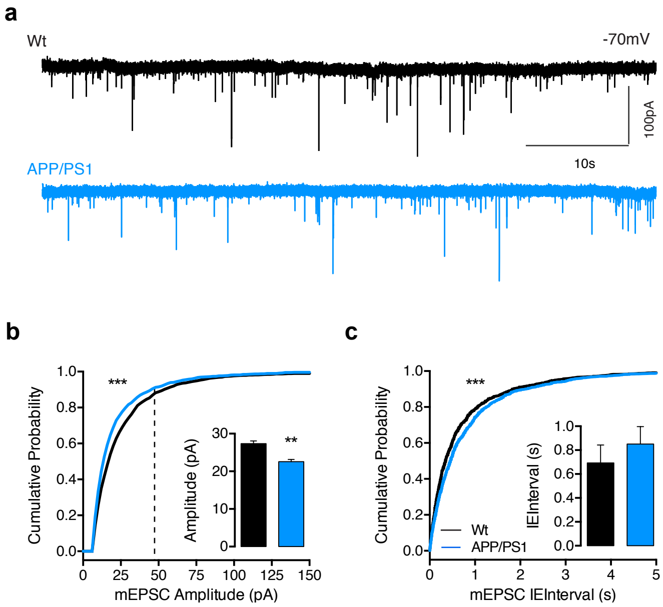

Supplementary Figure 2

(a) Representative recordings of mEPSCs obtained from CA3 PCs from Wt and APP/PS1 mice. CA3 PCs were held at -70 mV in the presence of bicuculline (10 μM) and TTX (1 mM). **(b)** Cumulative probability analysis of mEPSCs amplitude showed a difference between Wt and APP/PS1 mice (KS test $p < 0.0001$); the first 100 events of 15 cells per genotype are plotted. Average amplitude of mEPSCS is 28.7 ± 1.8 pA in Wt mice (n = 15 cells, 8 mice) and 22.6 ± 1.3 pA in APP/PS1 mice (n = 15 cells, 9 mice). **(c)** Cumulative probability analysis of mEPSCs inter-event interval (IEI) showed a difference between Wt and APP/PS1 mice (KS test $p<0.0001$); the first 100 events of 15 cells per genotype are plotted. Nevertheless the average frequency of mEPSCs is not different between Wt mice (0.7 ± 0.2 s IEI, n = 15 cells, 8 mice) and in APP/PS1 mice (0.9 ± 0.1 s IEI, n = 15 cells, 9 mice).

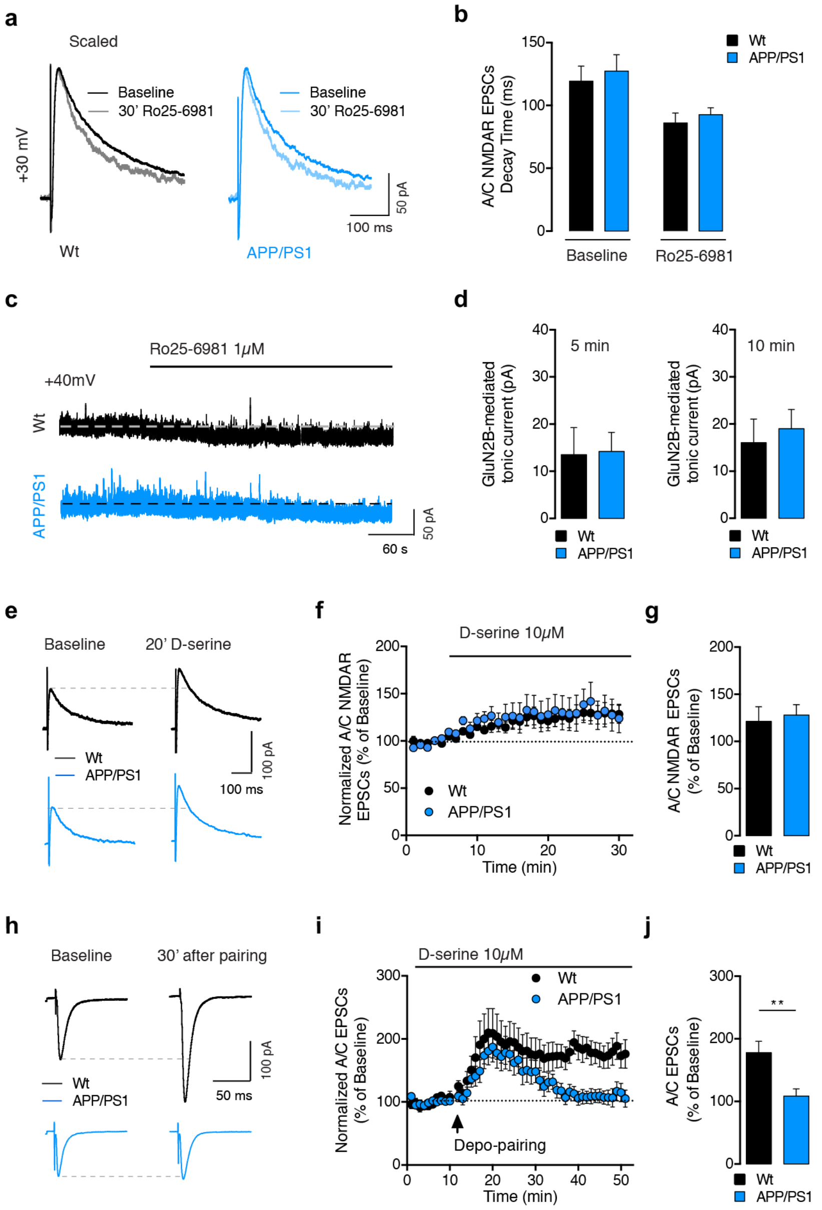

Supplementary Figure 3

(a) Average traces representing alterations in the decay time of A/C NMDAR-EPSCs after 30 min of Ro25-6981 action: darker traces correspond to an average during baseline period and lighter traces (scaled) to the average after 30 min of drug action. **(b)** Summary plot of the mean decay time of A/C NMDAR-mediated EPSCs shows that the effect of 1 μM Ro25-6981 ($p = 0.002$, two-way ANOVA with repeated measures (RM)) is not different between Wt mice ($n = 13$) and in APP/PS1 mice ($n = 16$, $p = 0.488$, two-way ANOVA RM and Sidak's multiple comparisons test). Accordingly, Ro25-6981 application reduced the decay time in Wt mice ($Wt_{Baseline}$ 119.3 ± 11.9 ms, $Wt_{Ro25\text{-}6981}$ 86.0 ± 7.9 ms, paired t-test $p = 0.035$; $APP/PS1_{Baseline}$ 127.3 ± 13.1 ms; $APP/PS1_{Ro25\text{-}6981}$ 92.7 ± 5.4 ms, paired t-test $p = 0.025$). **(c)** Representative examples of the shift in holding current observed at +40 mV in response to Ro25-6981

revealing the tonic activation of extrasynaptic GluN2B-containing NMDAR. **(d)** Summary bar graph of the extrasynaptic currents measured in (c) from CA3 PCs from Wt (n = 10) and APP/PS1 mice (n = 8, p = 0.927, Mann-Whitney test). Data calculated as the difference between last min of baseline and the last of 6 or 11 min after Ro25-6981 application. **(e)** Representative traces of A/C NMDAR-EPSCs measured at baseline and 20 min after D-serine application. **(f)** Summary plot describing the time course of A/C NMDAR-EPSCs potentiation after bath application of D-serine (10 μM). A/C NMDAR-mediated EPSCs (recorded at 0.1 Hz) were normalized to the mean amplitude of the baseline. **(g)** Bar graph representing a similar increase in mean amplitude of A/C NMDAR EPSCs recorded between 20 to 30 min after drug application. Values are expressed as percentage of the baseline amplitude and show no statistic difference between the effects of this NMDAR co-agonist on Wt or APP/PS1 synapses (Wt 121.3 ± 15.4 pA, n = 8; APP/PS1 127.0 ± 11.0 pA, n = 8; unpaired t-test p = 0.632). **(h)** Representative traces of average A/C EPSCs obtained during baseline and 30 min after the depo-pairing LTP protocol. **(i)** Summary time course of A/C LTP in the presence of D-serine. The LTP protocol was applied at the time indicated by the arrow. Slices were incubated with D-serine for 10 min before baseline recording and A/C AMPAR-mediated EPSCs were normalized to the mean amplitude of baseline. **(j)** Bar graph representing the effect of D-serine on A/C LTP. The LTP amplitude obtained in A/C synapses is different between APP/PS1 mice and Wt littermates (n = 11, **p = 0.008, unpaired t-test). D-serine did not rescue LTP in CA3 PCs of APP/PS1 mice (Supplementary Table 1). All electrophysiology recordings were performed in the presence of 10 μM bicuculline and 3 μM CGP55845.

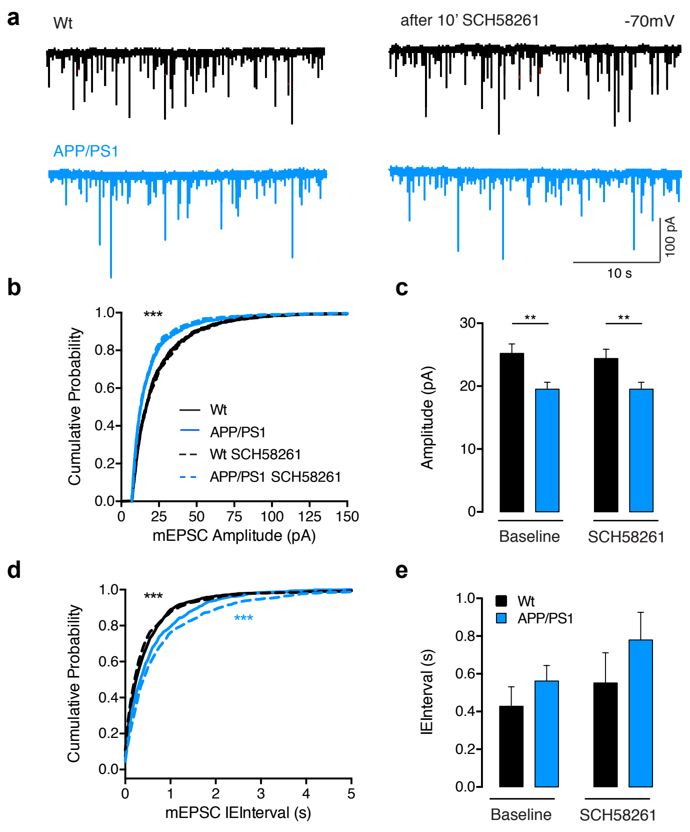

Supplementary Figure 4

(a) Representative recordings of mEPSCs obtained from CA3 PCs from Wt (n = 11 cells) and APP/PS1 mice (n = 11 cells) before and after a 10 min incubation period with SCH58261 (50 nM). CA3 PCs were held at -70 mV in the presence of bicuculline (10 μM), CGP55845 (3 μM) and TTX (1 mM). **(b)** Cumulative probability analysis of mEPSCs amplitude showed a difference between Wt and APP/PS1 mice (KS test ***$p < 0.0001$). For each cell, the last 100 events of a 10 min baseline period and the first 100 events after SCH58261 incubation are plotted. SCH58261 did not alter the amplitude's distribution of mEPSCs from both genotypes. **(c)** Summary bar graph of the average amplitude of mEPSCs during baseline and after SCH58261 incubation. Two-way ANOVA shows a genotype effect (**$p = 0.006$) while drug infusion has no effect. **(d)** Cumulative probability analysis of mEPSCs inter-event interval (IEI) showed an increase of IEI in APP/PS1 mice (KS test ***$p < 0.0001$). From each cell, the last 100 events of a 10 min baseline period and the first 100 events after SCH58261 incubation are plotted. Application of SCH58261 affected the distribution of mEPSCs IEI of APP/PS1 cells (KS test ***$p < 0.0001$) but had no effect on Wt cells. **(e)** Summary bar graph of the average IEI of mEPSCs during baseline and after SCH58261 incubation. Two-way ANOVA did not show statistical difference between genotypes or a drug effect.

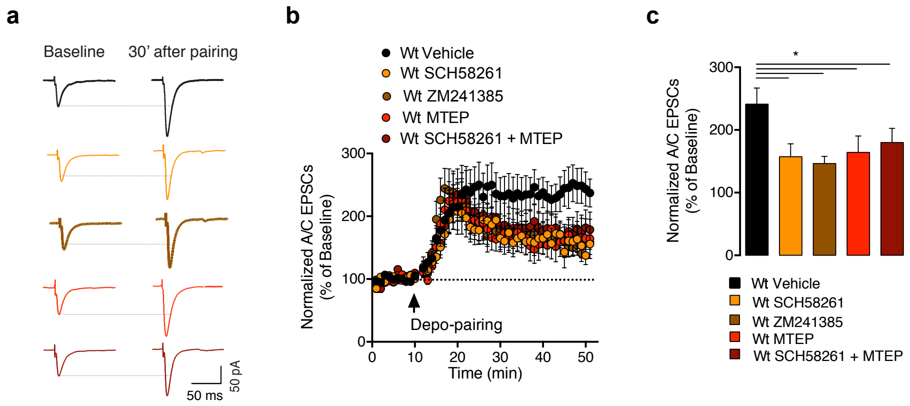

Supplementary Figure 5

(a) Example traces representing the average of A/C-EPSCs 10 min before and 30 min after depo-pairing LTP protocol in Wt animals in the presence of different antagonists of $A_{2A}R$ or mGluR5. **(b)** Summary time course of normalized A/C-EPSCs recorded from Wt mice during LTP protocol performed in the presence of either SCH58261 (50 nM), ZM241385 (50 nM), MTEP (10 μM) or SCH58261 and MTEP together. **(c)** Bar graph representing the mean LTP amplitude recorded from 30 to 40 min after depo-pairing protocol represented in (b). Incubation for 10 min of Wt mice slices with SCH58261 (n = 8), ZM241385 (n = 9), MTEP (n = 12) or MTEP + SCH58261 (n = 10) reduced the A/C LTP levels when compared with Wt vehicle (n = 9, p = 0.023, Kruskal Wallis test with Dunn's multiple comparison test between each group and $Wt_{vehicle}$). See detailed values in Supplementary Table 1. All recordings were performed in the presence of 10 μM bicuculline and 3 μM CGP55845.

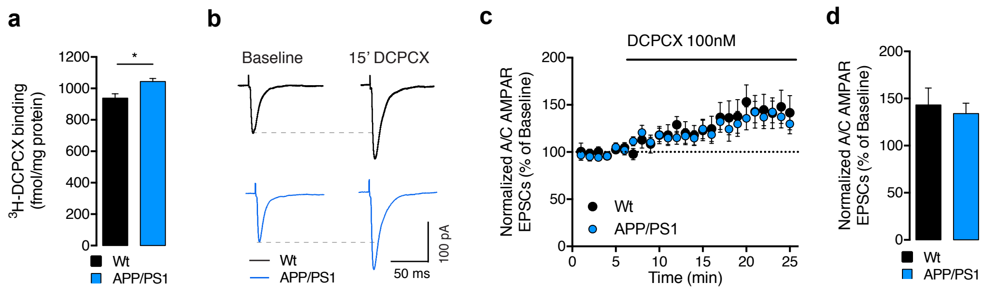

Supplementary Figure 6

(a) The density of A_1R shows a 10% increase in synaptic membranes prepared from the CA3 region of APP/PS1 mice compared to Wt littermates (n = 5, **p = 0.016, Mann-Whitney test). **(b)** Average traces of A/C AMPARs-EPSCs obtained at a stimulation frequency of 0.1 Hz during baseline and after 15 min from DCPCX (100 nM) application. Bicuculline (10 μM) and CGP55845 (3 μM) were present in the bath. **(c)** Summary plot of the time course of A/C AMPAR-EPSCs amplitude during the application of DCPCX in slices from Wt (n = 10) and APP/PS1 mice (n = 10). **(d)** Bar graph representing the similar increase of A/C AMPAR-EPSC amplitude after DCPCX application (p = 0.664, unpaired t-test). The amplitude of A/C AMPAR-mediated EPSCs was normalized to the mean amplitude of a 5 min baseline period and quantification was performed after 15 min of drug application.

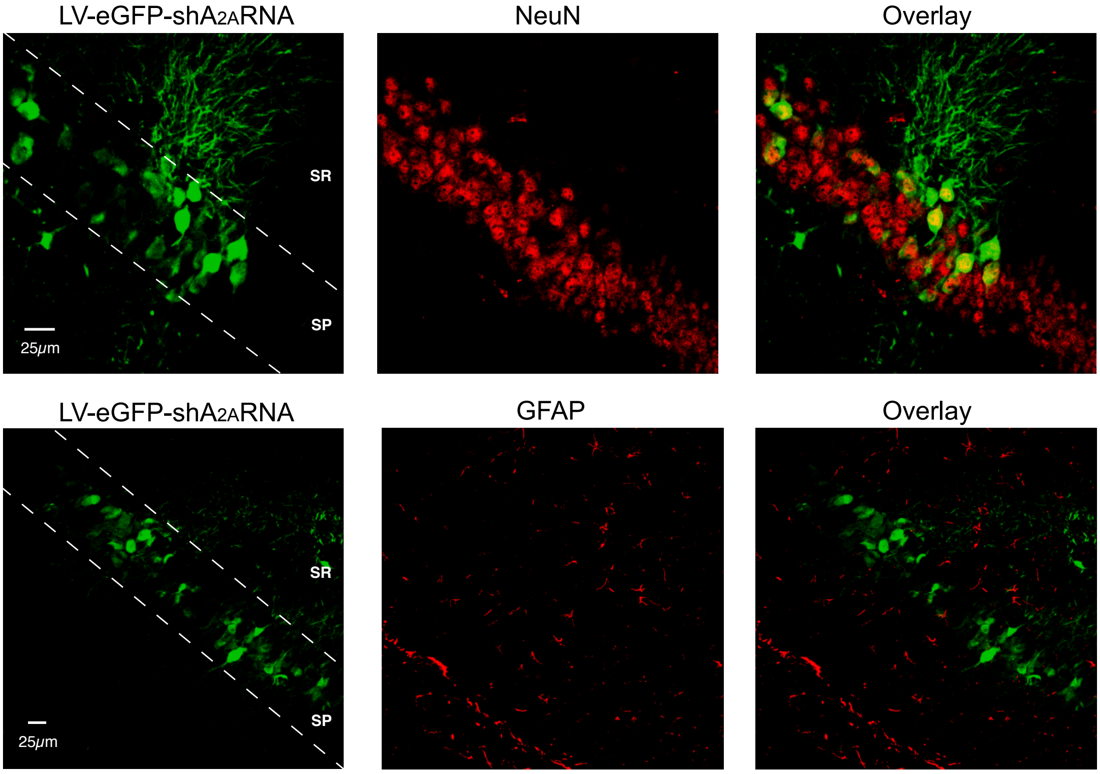

Supplementary Figure 7

Representative confocal images after immunohistological labeling (red) with either the neuronal marker NeuN, or with the astrocytic marker GFAP, used to test the tropism of $shA_{2A}RNA$ lentiviral vectors also expressing eGFP (green). Immunolabeling in the hippocampal CA3 region showed an almost exclusive colocalization of NeuN and virally infected eGFP positive cells (yellow, upper raw). No colocalization was found between GFAP and eGFP infected cells (bottom raw). The data are representative of 3 Wt mice analyzed. SP: *stratum pyramidale*, SR: *stratum radiatum*.

SUPPLEMENTARY TABLES

Supplemental Table 1 (related to Figures 1, 4, 6, 8, Supplementary figures 3 and 4)

Genotype	Treatment*	n	LTP level (%) (30 – 40min)	SEM	LTP?	p value
Wt	-----	11	224.9	27.9	Yes	0.005
APP/PS1	-----	10	90.7	9.8	No	0.432
Wt	D-serine	11	177.8	18.3	Yes	0.001
APP/PS1	D-serine	13	108.5	11.4	No	0.735
Wt	SCH58261	8	157.4	20.7	Yes	0.016
APP/PS1	SCH58261	11	159.7	15.5	Yes	0.003
Wt	ZM241385	9	146.4	11.3	Yes	0.008
APP/PS1	ZM241385	12	165.9	20.8	Yes	0.002
Wt	MTEP	12	164.3	26.1	Yes	0.002
APP/PS1	MTEP	12	163.6	27.9	Yes	0.016
Wt	SCH + MTEP	10	179.9	22.8	Yes	0.002
APP/PS1	SCH + MTEP	11	162.5	18.3	Yes	0.005
Wt	Vehicle (DMSO)	9	241.1	25.8	Yes	0.004
APP/PS1	Vehicle (DMSO)	11	98.2	8.6	No	0.520
APP/PS1	$shRNA^-$	7**	92.1	9.2	No	0.844
APP/PS1	$shRNA^+$	7**	173.7	20.3	Yes	0.008
APP/PS1	$scrRNA^+$	9	104.9	8.0	No	0.734
APP/PS1	$scrRNA^+$ + SCH	7	168.1	6.3	Yes	0.025
APP/PS1	$shRNA^+$ + SCH	9	173.4	14.7	Yes	0.002

Table 1 – Presence of LTP was evaluated by within-cell comparisons made with Wilcoxon match pairs test in raw non-normalized values between baseline values (average of 10 min) and the values obtained after applying the LTP protocol (30-40 min).

* all drugs were applied 5-10 min prior whole-cell establishment and kept for the rest of the experiment. All drugs were diluted in DMSO (100 mM stocks).

** n = 9 until 30 min after LTP (20-30 min). 4 pairs of cells ($shRNA^-$ and $shRNA^+$) were from the same animal.

Supplementary Table 2 (related to Figure 2)

	Range	Mean	Median	SEM	n
**Wt**					
spine length (μm)	0.19 – 1.81	0.82	0.77	0.02	300 spines<br>4 mice
head width (μm)	0.19 – 1.06	0.44	0.40	0.01	
neck length (μm)	0.05 – 1.38	0.46	0.40	0.01	
neck width (μm)	0.07 – 0.26	0.137	0.133	0.001	
head volume* (μm^3)	0.003 – 0.26	0.029	0.022	0.002	
CF	0.025 – 25.82	1.35	0.66	0.13	
**APP/PS1**					
spine length (μm)	0.23 – 2.12	0.81	0.75	0.02	288 spines<br>4 mice
head width (μm)	0.19 – 1.06	0.49	0.46	0.01	
neck length (μm)	0.09 – 1.33	0.40	0.35	0.01	
neck width (μm)	0.06 – 0.27	0.151	0.151	0.002	
head volume* (μm^3)	0.003 – 0.2	0.039	0.030	0.002	
CF	0.032 – 11.05	1.35	0.76	0.10	

Table 2 – Detailed information for each spine parameter measured.

*Head volume was calculated from the major and minor axes of the spine head dimensions assuming a prolate spheroid. CF = compartimentalization factor

Supplementary Table 3

Compound	Supplier	Activity	Concentration
Bicuculline	Tocris/R&D	Competitive $GABA_AR$ antagonist	10 μM
CGP 55845	Tocris/R&D	Competitive $GABA_BR$ antagonist	3 μM
NBQX	Abcam	Competitive AMPAR/KAR antagonist	20 μM
D-APV	Abcam	Competitive NMDAR antagonist	50 μM
L-CCG-I	Tocris/R&D	Potent group II mGluR agonist	10 μM
TTX citrate	Tocris/R&D	Na^+ channel blocker	1 μM
Ro 25-6981	Abcam	Blocker of $GluN2_B$ containing NMDAR	1 μM
SCH58261	Tocris/R&D	Competitive $A_{2A}R$ antagonist	50 nM
MPEP	Abcam	Non-competitive mGluR5 antagonist	10 μM
ZM241385	Abcam	Selective $A_{2A}R$ antagonist	50 nM

Table 3 – List of compounds and corresponding concentration used in all electrophysiology experiments.
